# Natural Products and Synthetic Analogs as a Source of Antitumor Drugs

**DOI:** 10.3390/biom9110679

**Published:** 2019-11-01

**Authors:** Javad Sharifi-Rad, Adem Ozleyen, Tugba Boyunegmez Tumer, Charles Oluwaseun Adetunji, Nasreddine El Omari, Abdelaali Balahbib, Yasaman Taheri, Abdelhakim Bouyahya, Miquel Martorell, Natália Martins, William C. Cho

**Affiliations:** 1 Zabol Medicinal Plants Research Center, Zabol University of Medical Sciences, Zabol 61615-585, Iran; 2 Graduate Program of Biomolecular Sciences, Institute of Natural and Applied Sciences, Canakkale Onsekiz Mart University, Canakkale 17020, Turkey; ademozleyen@gmail.com; 3 Department of Molecular Biology and Genetics, Faculty of Arts and Science, Canakkale Onsekiz Mart University, Canakkale 17020, Turkey; 4 Applied Microbiology, Biotechnology and Nanotechnology Laboratory, Department of Microbiology, Edo University, Iyamho, Edo State 300271, Nigeria; adetunji.charles@edouniversity.edu.ng; 5 Laboratory of Histology, Embryology and Cytogenetic, Faculty of Medicine and Pharmacy, Mohammed V University, Rabat 10100, Morocco; nasrelomari@gmail.com; 6 Laboratory of Zoology and General Biology, Faculty of Sciences, Mohammed V University, Rabat 10106, Morocco; balahbib.abdo@gmail.com; 7 Phytochemistry Research Center, Shahid Beheshti University of Medical Sciences, Tehran 1991953381, Iran; taaheri.yasaman@gmail.com; 8 Department of Pharmacology and Toxicology, School of Pharmacy, Shahid Beheshti University of Medical Sciences, Tehran 11369, Iran; 9 Laboratory of Human Pathologies Biology, Department of Biology, Faculty of Sciences, and Genomic Center of Human Pathologies, Faculty of Medicine and Pharmacy, Mohammed V University, Rabat 10106, Morocco; 10 Department of Nutrition and Dietetics, Faculty of Pharmacy, University of Concepcion, Concepcion 4070386, Chile; 11 Universidad de Concepción, Unidad de Desarrollo Tecnológico, UDT, Concepcion 4070386, Chile; 12 Faculty of Medicine, University of Porto, Alameda Prof. Hernâni Monteiro, 4200-319 Porto, Portugal; 13 Institute for Research and Innovation in Health (i3S), University of Porto, 4200-135 Porto, Portugal; 14 Department of Clinical Oncology, Queen Elizabeth Hospital, Hong Kong, China

**Keywords:** phytochemical, phytomedicine, anticancer, natural products

## Abstract

Cancer is a heterogeneous disease and one of the major issues of health concern, especially for the public health system globally. Nature is a source of anticancer drugs with abundant pool of diverse chemicals and pharmacologically active compounds. In recent decade, some natural products and synthetic analogs have been investigated for the cancer treatment. This article presents the utilization of natural products as a source of antitumor drugs.

## 1. Introduction

An abnormal development of cells that promulgates through the splitting of unrestricted cells is referred to as cancer. Cancer has been highlighted as one of the major issues of concern most especially for the public health system globally, and the US National Cancer Institute have forecasted with up rise of 50% in cancer cases, which will drastically increase to 21 million new cases in approximately two decades to this period [1]. According to the projection, it is likely to record seven out of ten deaths as a result of cancer in Central and South America, Asia, and Africa. This is an alarm that most developing countries need to upgrade and develop more strategic planning that borders around the issues of surveillance, early detection, and operational treatment for cancer patients [2]. Normally, individuals at a different time of their ages may be affected by cancer but there is a probability that the cancer diseases increases with increase in age [3]. This might be due to the accumulated DNA damage and multi-stage carcinogenesis as one becomes older [4,5,6].

The traditional approaches for cancer treatment include surgery, radiotherapy and chemotherapy [7,8]. Conversely, irrespective of the numerous type of synthetic drugs that have been utilized for the cancer chemotherapy and the healing accomplishment of various management schedules, the prevailing therapies have not yielded the level of expected result as tumor relapse and the beginning of metastasis often take place [9,10]. In light of this, there is a need to pursue more selective active compounds that have fewer side effects, are cost-effective, have more medicinal attributes, and have a minimum level of disease resistance have been enlisted as a major significant attribute necessary for cancer treatment most especially from biological and natural sources. Nevertheless, little information exists regarding the utilization of biological natural compounds and their cellular and molecular modes of action against cancer diseases. The late diagnosis and non-responsive therapy is a major reason of higher mortality among many cancer patients. This has instigated the need to search for an alternative cancer drug. The application of natural synthetic moieties is lead molecules that are preferable to some chemodrugs due to their various uncountable side effects [11,12].

The etiological process involved in the multiplying of a cancerous cell has been observed to be facilitated by some pathways or some mechanisms of action [13,14]. It has been stated that some active compounds derived from natural sources could be used effectively as a therapeutic technique for the treatment of these cancerous cells [15]. To date, more than 60% of synthetic drugs are derived from natural sources, out of which natural active compound most especially from plant constitutes 75% of anticancer drugs [16,17]. The natural product obtained from different sources portends the capability to enhance numerous physiological pathways which are necessary for the treatment of stalwart diseases [18] including cancer [19]. Therefore, it has become imperative to apply various strategies for the management of these stubborn diseases using natural products, most especially phytochemicals from biological sources most especially from plants [20,21]. This might be linked to the involvement of some crucial phytochemicals that have the capability to inhibit many pathways and prevent some malignancies, as well as the crucial roles they play in the inhibition of these dangerous cancer cells [22].

Nature is a rich source of anticancer drugs that are obtained from natural sources [23]. This might be linked to their abundant pool of diverse chemotypes and pharmacologically active. Moreover only a small percentage from these biologically active compounds derived from natural products have been formulated into clinically active drugs but their active compounds could be a model that will be followed for the formulation of more effective analogs and prodrugs through the utilization of chemical techniques like metabolomics, total or combinatorial fabrication or alteration of their biosynthetic pathways.

Furthermore, recent advances in formulating consortium of novel biologically active compounds may lead to may result in more efficient administration of the drug to patients. This might also include the fusion of toxic natural molecules to monoclonal antibodies and polymeric carriers precisely directed towards epitopes on the targeted tumor cell, which could result in the discovery of more active antitumor drugs. This also requires the inputs of multidisciplinary collaborations among different scientists to optimize and standardized the most active biological compounds and their adequate effectiveness as antitumor drugs at the molecular level [24,25,26,27,28].

For the past 10 years, some natural products have been discovered as a major source of drugs which have been utilized for the management of cancer chemotherapy, whereas almost 70% of them have been validated in various stages of clinical trials [16,17,29,30]. Some examples of antitumor drugs derived from plant compounds are Curcumin (diferuloylmethane) [31,32,33,34] and Paclitaxel (Taxol^®^), which is a taxane dipertene present in the crude extract obtained from the bark of *Taxus brevifolia* Nutt. (Western yew) [35]. Another example is Taxol (essentially all taxanes), which hinders microtubule disassembly by joining the microtubules that have been polymerized [35,36,37,38,39]. The bioavailability of these compounds is usually discussed, i.e., in the case of curcumin low bioavailability is addressed by using higher concentrations within nontoxic limits and its combination with other compounds or as formulations [40].

Examples of antitumor synthetic analogs derived from plant, which have been validated scientifically, include paclitaxel (Taxols) and the analogs docetaxel (Taxoteres) and cabazitaxel (Jevtanas); camptothecin and analogs belotecan (Camptobells), topotecan (Hycamtins), and irinotecan (Camptosars); vinblastine (Velbans), vincristine (Oncovins), and their analogs vindesine (Eldisines) and vinorelbine (Navelbines); and podophyllotoxin and analogs etoposide (Etopophoss) and teniposide (Vumons). Moreover, bacterial derived from the soil have also shown a lot of potential as a great source of antitumor drugs like the glycopeptide bleomycin (Blenoxanes), the nonribosomal peptide dactinomycin (Cosmegens), anthracyclines doxorubicin (Doxils; Adriamycins) and daunorubicin (Cerubidines), and epirubicin (Ellences) (Table 1) [17,24,41].

The compact and unusual structural configuration of some of these natural compounds plays a crucial role in their joining together to specific targets or molecular interfaces. This might result in some level of phenotypic alteration, most especially in biological systems that involve fixing of natural molecules, entails structural requirements that allow their binding to specific targets or molecular interactions [42,43]. Some of these features shared the same similar medicinal attributes in the most different diseases. It has been validated that 64% of drugs derived from natural products are effectually used in the development of these drugs [17,44].

Moreover, due to the invaluable biological diversity of natural products, there is a need to still search for more effective antineoplastic activity active compounds from microorganisms, most especially from marine sources and from unexploited plants because some of these compounds might show exceptional activities when tested against new medicinal targets [41,45]. Examples of marine-derived anticancer drugs include the conjugated antibody brentuximab vedotin (Acentriss), cytarabine (Cytosars), eribulin mesylate (Halavens), and trabectedin (Yondeliss) [46,47,48].

Therefore, this review presents a holistic view of the current trends towards the utilization of natural products and synthetic analogs as a source of new antitumor drugs that have been reported for the past decade. Moreover, recent information about antitumor drugs derived from various sources and their general bioactivity towards the management of different types of cancer is well elaborated in this review work.

## 2. Antitumor Drugs: A Brief Medical History, Different Origins, and General Bioactivity

Cancer has been reported as the second most common cause of death with an estimated 9.6 million deaths in 2018 by the World Health Organization [49]. It is not an emerging disease: people have been suffering from cancer throughout the world for centuries. Between 460 and 370 B.C, Hippocrates used the word cancer for the first time to describe carcinoma tumors [50]. However, this disease is not discovered by Hippocrates. The pieces of evidence showed that bone cancer was reported in ancient Egypt mummies in approximately 1600 B.C. and breast cancer in 1500 B.C.; however, there was no recorded treatment for cancer [51].

Considering the earliest reports on the nature of cancer, first findings dates back to 1761, when Giovanni Battista Morgagni, regarded as the father of modern anatomical pathology, did autopsies for the first time on dead bodies to elucidate the relation between patient’s illness and pathologic observations. Giovanni’s studies provided the basis of scientific cancer strategies [52]. Additionally, John Hunter who introduced the idea that surgery could be a strategy for the patients whose tumors have not invasive and moveable characteristics to nearby sites, he said: “there is no impropriety in removing it.” [53]. A century later, anesthesia was invented, and surgeons Bilroth, Handley, and Halsted carried out cancer operations by removing the entire tumor. Development of modern microscope accelerated the studies in the era of scientific oncology in the 19th century and Rudolf Virchow, the founder of cellular pathology, laid the foundation of the modern pathologic study of cancer [54]. Thus, damages caused by cancer on the body could be detected. Moreover, the efficiency of operations could be examined by this method whether the cancerous tissue had been completely removed from the cancer site [54].

Early in the 20th century, surgery and radiotherapy were the two most dominated modalities to cure cancer diseases [55]. The term ‘‘chemotherapy’’ was provided into literature by the famous German chemist Paul Ehrlich in the early 1900s. It means the therapeutic use of chemicals to treat diseases [56]. He was also the first scientist who evaluated the potential biological activities of a group of chemicals in animal models. He is a pioneer to overcome the major limitation in the cancer drug development process before clinical stages [56,57].

**Figure 1:**
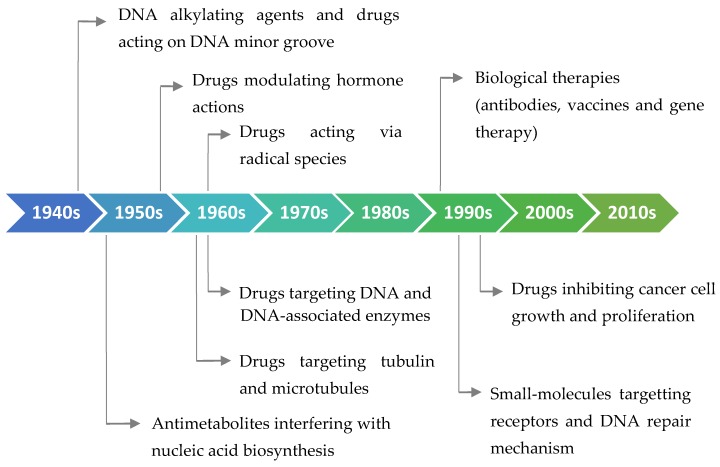
Classification of the antitumor drugs according to their action mechanism and timeline showing their history.

In the middle of the 20th century, during the World War II, breakthrough information emerged in the field of chemotherapy. It was reported that people exposed to mustard gas in the field of military action had toxic changes in the bone marrow cells [57,58]. As a result of these surprising findings, researchers focused on the mustard gas-related compounds to identify effective compounds to cure cancer. After much effort on their part, the first anticancer drug, called mechlorethamine (Mustargen^®^), was approved for the treatment of lymphoma and reached the markets in 1949 as a nitrogen mustard alkylating agent [57,59,60]. The discovery of nitrogen mustard paved the way for the synthesis of other anticancer drugs. Sidney Farber, in 1948, showed the effectiveness of aminopterin, a folic acid antagonist, against childhood leukemia and it was the predecessor of the drug methotrexate that is still utilized in clinics [58]. When the historical development of antitumor agents has been examined, it can be seen that drugs that were discovered in the second half of the 20th century have generally exerted their effects through direct binding to DNA thus creating cell toxicity (Figure 1). However, in the 21st century, with the advent of molecular biology techniques, the action mechanism of chemotherapeutic agents has become quite specific. The trend has been changing from small molecules to protein-based therapeutics as well as their small molecules conjugated forms (Figure 1).

These findings were significant milestones in anticancer drug developments which result in an increased number of drugs reaching to the markets [60]. Between 1950 and 1980, on average, two new drugs were approved for their anticancer activities a year. Moreover, this number was doubled in the 1990s. An average of 10 novel oncologic drugs hit the markets in the years between 2011 and 2019 per year [59]. Unlike the drug development process in the past, it takes ~13–15 years for the validation of drugs in the preclinical and clinical phases. There are several stages in this workflow, including determination of molecular and phenotypic targets; design, in silico analysis, and synthesis of hit molecules; in vitro and animal studies for the elucidation of biological effects of test compounds; and optimization of a candidate compound for clinical studies. The efficacy, safety, and possible side effects of drugs are tested through clinical phases [61]. In the following sections, the latest drugs approved by the Food and Drug Administration (FDA) USA in the last decade are summarized according to their mechanism of action. Nearly, 80% of FDA-approved drugs during the last three decades for cancer treatment are either natural products per se or derivatives [62].

### 2.1. FDA-Approved Small Molecules as Antitumor Drugs in the Last 10 Years

In the last decade, more than fifty small molecules as antitumor agents have been approved by the FDA (Table 2).

**Figure 2:**
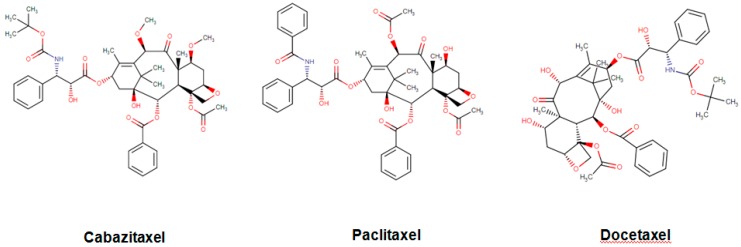
Chemical structures of cabazitaxel, paclitaxel and docetaxel.

Among these drugs, cabazitaxel (Jevtana) is a second-generation semisynthetic taxane derivative approved in 2010 by the FDA, especially for the treatment of metastatic hormone-refractory prostate cancer. Taxanes are a class of diterpenes, which were originally identified from plants of the genus Taxus (yews). Paclitaxel (Taxol) and docetaxel (Taxotere) are widely used progenitor of cabazitaxel (Figure 2). Their mechanism of action involves microtubule stabilization to induce cell death. In general, they bind to tubulin subunits and promote the assembly of microtubules while simultaneously inhibiting its disassembly. This leads to arrest in the cell cycle at the metaphase and triggers apoptosis in the cancerous cell. Although they have a similar mechanism of action, cabazitaxel has an advantage over paclitaxel and docetaxel, due to having extra methyl groups that mitigate constitutively and acquired antitumor drug resistance by inhibiting the P-glycoprotein (P-gp) efflux pump [63,64]. Besides, cabazitaxel is more effective in central nervous system (CNS) metastases because of its ability to pass through the blood–brain barrier [64].

**Figure 3:**
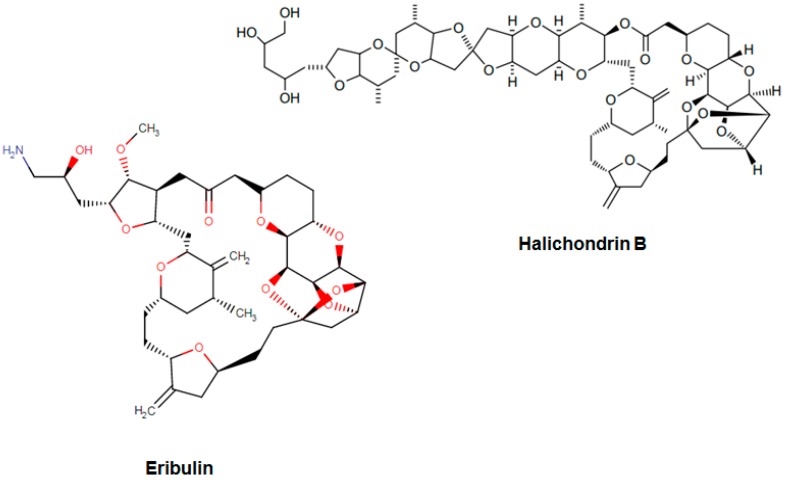
Chemical structures of eribulin and halichondrin B.

Eribulin (Havalen) was isolated from the marine sponge *Halichondria okadai*. It is a non-taxane microtubule inhibitor with a novel mode of action and was approved by FDA in 2010 for the treatment of patients with metastatic breast cancer who have previously administered with at least two chemotherapeutic protocols. Although it is a fully synthetic organic molecule, its structure was inspired by halichondrin B, which is a polyether macrolide isolated from the rare marine sponge in 1986 by Hirata and Nemura [65]. The studies with halichondrin B had reported a remarkable microtubule-associated in vivo and in vitro anticancer activity [66]. As a result of these findings, in 1992, the total synthesis of halichondrin B was achieved by Kishi and colleagues [67]. Several studies with this compound revealed that macrocyclic lactone C1–C8 moiety on the right half of the molecule retains its cytotoxic activity [67]. Although Eribulin is a structurally much simpler analog of halichondrin B, it retains a biologically active pharmacophore of the original molecule (Figure 3) [68,69,70].

The cytotoxicity of eribulin is mediated through microtubules; however, its mode of action is different from other tubulin-binding agents such as taxanes and vinca alkaloids [71,72]. These agents bind along the sides of microtubules, specifically eribulin, and limitedly bind on the (+) ends of the structure, thus inhibiting polymerization but not depolymerization (shortening) of its growth. Therefore, eribulin results in the arrest of the cell cycle at the G_2_/M phase thus activation of the apoptotic processes and subsequently cell death [66,73,74,75].

**Figure 4:**
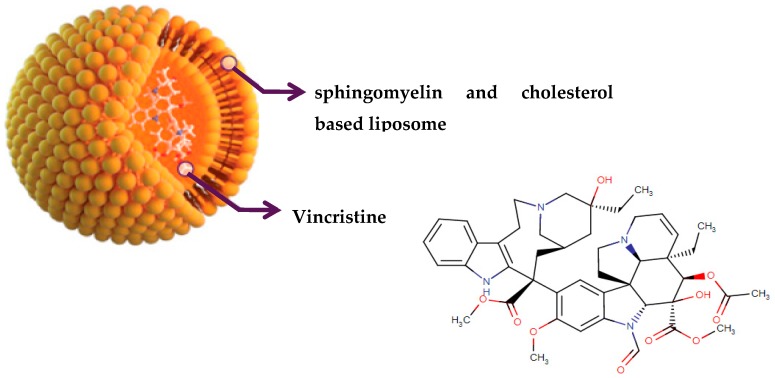
Chemical structure of vincristine and formulation of VinCRIStine sulfate liposome injection (Marqibo).

Another potent and widely used chemotherapeutic agent representing its antitumor activity through microtubule inhibition is vinciristine—a kind of plant alkaloid. It was first isolated from the extract of the periwinkle plant *Catharanthus roseus* (L.) G.Don (formerly known as *Vinca rosea* L.) in the scope of a screening program exploring the potential antidiabetic agents [76,77,78]. The action mode of vincristine includes microtubule depolymerization through binding to tubulin subunits resulting in metaphase arrest and finally apoptotic cell death. Although vincristine has been effectively used for more than 50 years in the treatment of hematologic malignancies and solid tumors, it has important limitations due to its suboptimal pharmacokinetic profiles and dose-related neurotoxicity [79]. VinCRIStine sulfate Liposome injection (Marqibo) is a novel and therapeutically improved formulation of vincristine encapsulated in sphingomyelin and cholesterol based nanoparticles. This liposome-remodeled form of the active compound is approved by FDA in 2013 for the treatment of relapsed Philadelphia chromosome-negative acute lymphoblastic leukemia (Figure 4).

**Figure 5:**
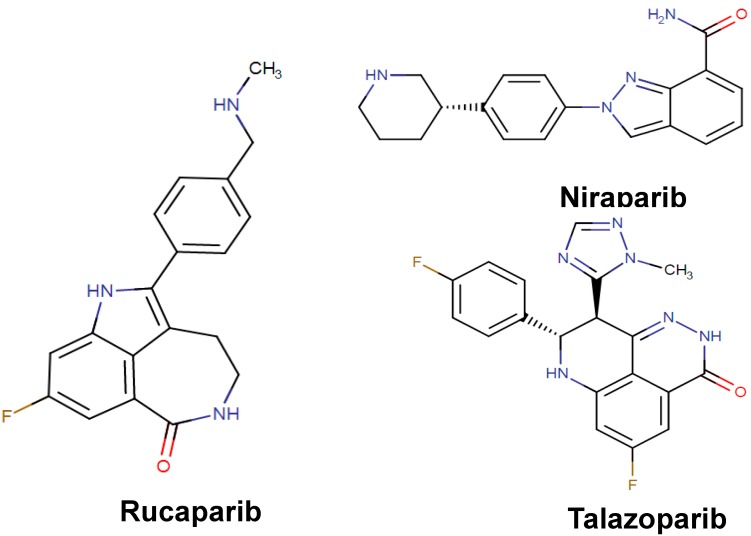
Chemical structures of rucaparib, niraparib, and talazoparib.

In the last decade, poly(ADP-ribose) polymerases (PARPs), which are ubiquitous zinc finger DNA-binding enzymes, have been established as well-known targets of several oncologic drugs The cellular roles of PARP proteins include regulation of homologous recombination, transcription, and replication processes, as well as DNA repair mechanism under any stress conditions [80]. Inhibition of the PARP proteins can bring about the stimulation of apoptotic pathways through NAD^+^/ATP depletion, loss of mitochondrial membrane dynamics, and the production of excess amount of apoptosis-inducing factor [80]. Recently, the drug rucaparib (Rubraca, Figure 5), found in the class of piperidine type organic compounds, has been approved by FDA (2016) as a potent PARP-1, -2, and -3 inhibitor for the treatment of advanced ovarian cancer in women with deleterious germline or somatic BRCA mutation [81]. Soon after, another piperidine compound, niraparib (Zejula, Figure 5), was approved by the FDA (2017) for its effectiveness on recurrent epithelial ovarian cancer [82]. The drug exerts its effect by specifically inhibiting PARP-1 and PARP-2 activation resulting in niraparib-induced cytotoxicity in cancerous cells. Very recently, a kind of quinoline derivative talazoparib (Talzenna, Figure 5) has been approved by FDA (2018) for use in the treatment of germline BRCA mutated, HER2-negative, locally advanced, or metastatic breast cancer due to its inhibitory effect on PARP-1 and PARP-2 proteins [83].

**Figure 6:**
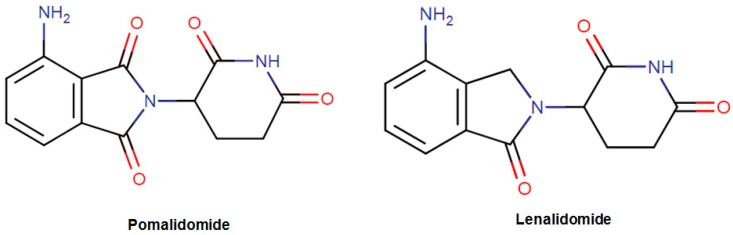
The structure of pomalidomide and lenalidomide.

Chronic inflammation refers to the uncontrolled immune response of living organisms to initiate a defense mechanism against several endogenous and exogenous stimuli [84,85,86]. However, a prolonged inflammatory response is associated with the production of an excess amount of reactive nitrogen/oxygen species, persistent cytokine release and sustained immune response [85,87]. This might lead to various inflammation-related pathological diseases including type II diabetes, coronary-neurologic disorders, as well as cancer [88,89]. The drug pomalidomide (Pomalyst; found in the family of organic compounds called phthalimides—Figure 6) was approved in 2013 by the FDA to be used in the treatment of patients having relapsed and refractory multiple myeloma [90]. It has been demonstrated that the drug is an immunomodulatory agent with multiple actions including the cytotoxic and apoptotic effects on tumor cells. As having immune modulatory effects, the drug is highly effective inhibitors of proinflammatory cytokines such as TNF-α, IL-6, and even transcription of COX2 [90]. It is thought the primary biological target of the drug is the protein cereblon to suppress ubiquitin ligase activity. In the same year, another drug called lenalidomide (Revlimid; found in the family of organic compounds known as isoindolones—Figure 6) was introduced into the markets for the treatment of mantle cell lymphoma. It has been shown that the drug inhibited the release of proinflammatory cytokines and increased the release of anti-inflammatory cytokines from peripheral blood mononuclear cells [91]. In addition to these effects, the drug inhibited the expression of COX-2 selectively but not COX-1. Additionally, the drug stimulated the apoptosis of tumor cells by the inhibition of bone marrow stromal cell support and immunomodulatory activity [92].

**Figure 7:**
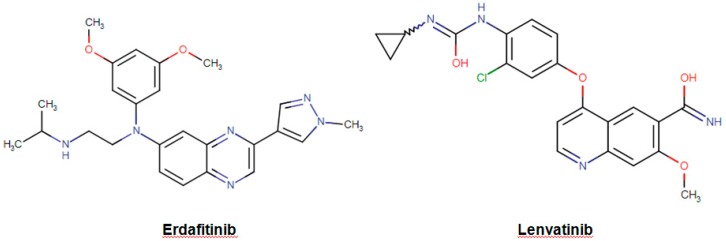
Chemical structures of erdafitinib and lenvatin.

Receptor tyrosine kinases (RTKs) are a group of cell surface receptors that are responsible for the regulation of cell growth, motility, differentiation, and survival [93]. The sustained activation and expression level of RTKs have been found to be correlated with the abnormalities in the downstream signaling pathways such as mitogen-activated protein kinase (MAPK), phosphoinositide 3-kinase/protein kinase B (PI3K/Akt) and Janus kinase/signal transducers, and activators of transcription (JAK/STAT) pathways, which may finally result in cancer development and uncontrolled proliferation of the cells [94,95]. Therefore, during the past decade, different domains of RTKs including extracellular, transmembrane, and cytoplasmic domains have been studied extensively as therapeutic targets in the development of anticancer agents [96]. The drug erdafitinib (Balversa; found in the class of organic compounds named alkyldiarylamines, Figure 7) is the latest FDA-approved (April 2019) oncologic drug as well as the first-ever fibroblast growth factor receptor (FGFR) kinase inhibitor for the treatment of patients who are suffering from advanced urothelial carcinoma [97]. In normal tissues, FGFR proteins ubiquitously expressed for the regulation of various physiological processes such as phosphate and vitamin D homeostasis, as well as proliferation and antiapoptotic signaling of the cells [98]. However, in the case of cancer, upon binding of FGF ligands to the receptor, the downstream signal transduction is stimulated leading to permanent activation of phosphoinositide phospholipase C (PLCγ), MAPK, AKT, and STAT cascades [98]. The drug erdafitinib is said to be a selective inhibitor for the tyrosine kinase enzymatic activities of expressed FGFR1, FGFR2, FGFR3, and FGFR4. The drug lenvatinib (Lenvima; an organic compound known as quinoline carboxamides, Figure 7) was approved by FDA in 2015 as a multipotent RTK inhibitor, which has been found to be effective in the treatment of patients with locally recurrent or metastatic, progressive thyroid cancer [99]. Lenvatinib inhibits the activities of several vascular endothelial growth factor (VEGF) receptors VEGFR1 (FLT1), VEGFR2 (KDR), and VEGFR3 (FLT4). The production of endogenous VEGF is necessary for some physiological processes such as fetal development, menstruation, and wound healing in normal tissues [100]. However, the overproduction of VEGF is associated with abnormal tumor growth and metastasis by stimulating the formation of new blood vessels from existing vasculature [101]. Lenvatinib exerts its effect by binding to the adenosine 5′-triphosphate site of VEGFR and to a neighboring region, leading to inhibition of tyrosine kinase activity as well as downstream cascades. Lenvatinib also inhibits other RTKs including fibroblast growth factor (FGF) receptors FGFR1, 2, 3, and 4; the platelet-derived growth factor receptor alpha (PDGFRα); KIT; and RET that have been implicated in the pathogenic angiogenesis, tumor growth, and cancer progression.

### 2.2. FDA-Approved Protein-Based Therapeutics in the Last 10 Years

Understanding the fundamental of proteins and novel techniques utilized in genetic engineering has made a great contribution to the field of the pharmaceutical industry. Unlike small molecule drugs, protein therapeutics cannot be produced via a sequence of chemical reactions, so natural sources, such as living cells or organisms, are essential hosts for the manufacture of a product [103,104]. Although protein-based drugs are highly target-specific and potent therapeutics, the characterization of final products is a highly challenging process because of their large molecular size, exhausting purification steps, as well as individual variations in cancer patients [105].

Similar to numerous small molecules, a group of protein-based therapeutics has been approved for their inhibitory effect on RTKs and downstream signaling pathways. Human epidermal growth factor receptor 2 (HER2) is a significant prognostic and predictive biomarker commonly researched in oncological clinics. The overexpression of the HER2 is much higher in the breast and gastric/gastroesophageal cancer patients [105]. Moreover, the HER2 positivity has been found to be associated with other cancer types such as ovary, endometrium, bladder, lung, as well as the colon [105]. Binding of ligands to the receptor leads to the autophosphorylation of tyrosine residues within the cytoplasmic domain after that dimerization of receptors resulting in cell proliferation, angiogenesis, and invasion-related signaling pathway activations [106]. Elucidation of the structure and other characteristics of HER2 have paved the way for a more effective personalized therapeutic strategy in HER2-positive patients. The drug trastuzumab (Herceptin; recombinant humanized IgG1 monoclonal antibody) has demonstrated high affinity against the extracellular domain of HER2, resulting in inhibition of cancer cells growth and proliferation, and increased survival in the breast and gastric cancer patients [107]. Additionally, the biosimilar drugs to Herceptin, named trastuzumab-dkst (Ogivri), trastuzumab-pkrb, (Herzuma), and trastuzumab-anns (KANJINTI), have been approved for their therapeutic effects in the patients with metastatic breast and gastric cancer.

As an alternative to small molecules that are able to inhibit the function of VEGF receptor, the protein-based drugs bevacizumab (Avastin; recombinant humanized IgG1 monoclonal antibody) and ramucirumab (Cyramza; human IgG1 monoclonal antibody) have been introduced for their pharmacological actions against blood vessel proliferation and metastatic tumor growth in the patients with cervical cancer and gastric cancer respectively [108,109]. Bevacizumab exerts its effect by binding to VEGF and preventing the interaction between VEGF and its receptors, named Flt-1 and KDR, found on the surface of endothelial cells. On the other hand, ramucirumab shows high affinity against VEGFR2 and prevents the ligand-induced proliferation of endothelial cells by limiting the interaction between ligands (VEGF-A, VEGF-C, VEGF-D) and VEGF receptor [108].

In 2018, the Nobel Assembly at Karolinska Institutet decided to award Tasuku Honjo jointly to James P. Allison in the category of Physiology or Medicine for their discovery of cancer therapy by inhibition of brake-like regulators found in the immune system [110]. T cells are a group of white blood cells that play a significant role in the defense mechanism of a living organism by trigger the immune system [111]. James P. Allison and other research teams have worked to elucidate the biological role of cytotoxic T lymphocyte-associated protein 4 (CTLA-4) in the treatment of several cancers and autoimmune diseases models [112,113,114]. After the activation of T cells, the expression of CTLA-4 is stimulated, which downregulates immune responses by binding to the cluster of differentiation 28 receptors. James P. Allison proposed that the CTLA-4 blockade could encourage T cells to fight cancer cells [113,114]. The animal and clinical studies gave promising results for the treatment of advanced melanoma, a type of skin cancer [115]. In line with these developments, the designed drug ipilimumab (Yervoy; humanized IgG1 monoclonal antibody) has been approved by FDA to be utilized for the 12 years and older patients with metastatic melanoma [116]. Its action mechanism is based on inhibition of the activity of CTLA-4, thereby sustaining the activation of T cells to fight against tumor cells.

Additionally, Tasuku Honjo discovered the protein known as programmed cell death protein 1 (PD-1) that is localized in the T cell surfaces. PD-1 proteins behave like a brake in immune response results in inhibition of T cell activation [117]. Interestingly, Honjo and his research group demonstrated that blockage of PD-1 could be an effective strategy for cancer treatment in the animal models. These promising results led to high attention on PD-1 as a biological target in pharmacological research [117].

The drug pembrolizumab (Keytruda; humanized IgG4-kappa monoclonal antibody) has been approved by FDA in 2014 for the treatment of patients with lung cancer, advanced renal cell carcinoma, breast cancer, metastatic cervical cancer, primary mediastinal B-cell lymphoma, as well as hepatocellular carcinoma [118]. It binds to PD-1 with high affinity, thus the interaction between its ligands (PD-L1 and PD-L2) and the receptor is prevented to maintain T cell proliferation and cytokine production. Moreover, another drug called nivolumab (Opdivo; human IgG4 monoclonal antibody) shows a higher affinity to immune checkpoint PD-1 to induce the natural tumor-specific T cell immune response of patients with metastatic colorectal cancer [119]. On the other hand, a number of approved drugs have been designed to block directly the PD-L ligands for the sustained T cell activation. Recently, the drug durvalumab (Imfinzi; human IgG1 kappa monoclonal antibody) has been designed specifically for programmed death ligand 1 (PD-L1) to block the receptor–ligand interaction. It exhibited a curative effect in patients with metastatic urothelial carcinoma and non-small cell lung cancer [120]. With the same action mechanism, atezolizumab (Tecentriq; Fc-engineered, humanized, monoclonal antibody) has been introduced for the treatment of locally advanced or metastatic urothelial carcinoma [121].

### 2.3. FDA-Approved Antibody–Drug Conjugates in the Last 10 Years

One of the latest improvements in chemotherapeutic strategies is combining a FDA-approved cytotoxic small molecule with an antibody directed to a specific protein found on the tumor cells. This strategy acts like a double-edged sword, allowing the specific targeting of the tumor cells while simultaneously delivering two potent cytotoxic agents [122,123]. Moreover, it provides preferable efficacy and reduced risk of systemic toxicity compared to the existing chemotherapy strategies [123]. Ado-trastuzumab emtansine (Kadcyla) is a kind of approved drug–antibody conjugate that is utilized for the treatment of HER2-positive metastatic breast cancer [124]. In this example, although the antibody compartment of the drug is HER2-specific humanized IgG1 (trastuzumab), which inhibits HER2 receptor signaling and leads to antibody-dependent cytotoxicity, the conjugated drug a maytansine derivative (DM-1) interferes with microtubules function, which results in cell cycle arrest and apoptosis [125]. Another drug, brentuximab vedotin (Adcetris), was approved by the FDA in 2011 for the treatment of patients with Hodgkin’s lymphoma and systemic anaplastic large cell lymphoma. However, one year later, it was revised with boxed warning due to post-treatment-based side effects and deaths. In March 2018, the FDA approved brentuximab vedotin to treat adult patients with previously untreated stage III or IV classical Hodgkin lymphoma. The drug combines an anti-CD30 human-murine IgG1 with the drug monomethyl auristatin E (MMAE). Antibody provides the detection of cancer cells expressing CD30 whereas conjugated drug MMAE targets microtubules and distrupts their structure.

The strategy of protein-drug conjugate also provides design of therapeutic agents with improved half-life, qualified pharmocokinetics as well as stability. The drug calaspargase pegol-mknl (Asparlas) has been approved for the treatment of acute lymphoblastic leukemia in pediatrics and young adults by the FDA in 2018 [126,127]. The drug contains *Escherichia coli*-derived enzyme L-asparaginase II and monomethoxy polyethylene glycol (pegol) linked by succinimidyl carbonate in its structure. The enzyme L-asparaginase converts the L-asparagine to L-aspartic acid, resulting in a sharp drop in the present asparagine concentration. This decrease blocks protein synthesis and tumor cell proliferation in the cancer tissue. Moreover, the conjugated pegol group decreases enzyme antigenicity and increases the half-life of the drug [127].

## 3. Plants and Related Bioactive Compounds as New Drug Sources for Different Cancers

### 3.1. Oral, Gastrointestinal, and Pancreatic Cancers

Since ancient times, in East Asia, traditional medicinal plants have been used to treat many diseases, including cancer [128,129,130]. In fact, in recent decades, despite the evolution of conventional treatments for oral cancer, morbidity and mortality rates have been steadily increasing. Indeed, the 5-year mortality rate is ~50% [131,132]. Recently, studies reported the anticancer effects of bioactive compounds from medicinal sources on oral cancer cell lines (Table 3). Nam, et al. [133] tested the anticancer activity of artemisinin and its various derivatives against oral cancer cell line (YD-10B) and they found as results an induction of apoptosis by the activation of caspase-3, which is the general mediator of apoptosis [134,135]. Several studies have evaluated the antitumor activity of eugenol in vitro against different oral cancer cells, and the results obtained showed that this compound had a good cytotoxic activity [136,137,138]. On the other hand, it has been shown that polyphenols also have anticancer properties [139]. Effectively, safrole, a polyphenolic compound of *Piper betle* L., has been tested in vitro on human buccal mucosal fibroblasts (BMFs) to prove its cytotoxic effect [140]. Additionally, phytochemicals reduce the risk of cancer [141]. Indeed, berberine, the main constituent of *Coptis chinensis* Franch., has been used in the treatment of gastrointestinal disorders including oral cancer cells (KB, OC2) [142]. In addition, it proved effective against HSS3 oral cancer cells by inducing apoptosis [143]. Thus, it has been reported that carvacrol possesses antitumor properties [144]. Sertel et al. studied the antitumor activity of three medicinal plants on UMSCC1 cells and found a significant cytotoxic effect [145,146,147]. While Manosroi et al. [148] found the same effect regarding essential oils (EOs) from 17 Thai medicinal plants against KB cells, followed by Cha et al. [149,150] and Keawsa-ard et al. [151], who evaluated the anticancer effect of *Artemisia gmelinii* Weber ex Stechm. (synonym of *Artemisia iwayomogi* Kitam.) and *Solanum spirale* Roxb., respectively, on human mouth epidermal carcinoma (KB). EOs of aromatic and medicinal plants have many biological activities [152,153]. Moreover, EOs derived from the herbal plant *Pinus densiflora* Siebold & Zucc. can induce apoptotic cell death via reactive oxygen species (ROS) generation and activation of caspases in YD-8 human oral cancer cells. Note that the accumulation of ROS, under the conditions of oxidative stress, causes tissue toxicity by modifying cellular macromolecules such as proteins, lipids and DNA, which irreversibly alter cell viability and function [154,155]. In another study using the KB cell line, *Artemisia capillaris* Thunb. EOs had an anticancer effect related to the induction of apoptosis and activation of the MAPK signaling pathway [149,150]. The isolated limonoides of *Azadirachta indica* A.Juss. have been studied by Harish Kumar et al. [156], who showed apoptosis on the hamster buccal pouch (HBP) carcinogenesis model. Finally, other compounds used in the treatment of oral cancer such as chalcone extracted from the medicinal plant *Alpinia pricei* Hayata [157] and deoxyelephantopine (ESD) isolated from *Elephantopus scaber* L. [158] have shown the ability to induce cell cycle arrest and apoptosis in human oral carcinoma HSC-3 cells and human nasopharyngeal carcinoma (CNE) cells, respectively.

On the other hand, several bioactive compounds found in medicinal and aromatic plants have shown antitumor properties against gastrointestinal cancer [159,160,161,162,163,164,165,166]. The results of these works are summarized in Table 4. Liang et al, [159] tested Ganoderma against HCT116 cell line and showed that this compound induced apoptosis in HCT116 and increased caspase-8, caspase-3, and Fas. Genistein exerted an important antiproliferative effect, proapoptotic effect in vitro HT-29 cell line by increasing the expression of Bax or p21 proteins, and inhibiting NF-kB and topoisomerase II expression [162,163]. The combination of this compound with cisplatin inhibited cell growth and induced apoptosis. On the other hand, the in vivo assay showed that some triterpenes are reported as antitumor drugs against HT-29 cell line via the suppression of proliferation and inhibition of tumor growth in the colon carcinoma xenograft model [160]. Ginkgo biloba has also exhibited an in vitro anticancer effect on HT-29 cell line by the inhibition of tumor progression, the increasing caspase-3 activity, the elevating p53 expression, and decreasing of bcl-2 expression. In another in vivo study, Romano et al [161] reported that cannabidiol exhibited an important anticancer activity on HCT116 mice xenograft through the reduction of the pre-neoplastic lesions and azoxymethane-induced polyps. Flavonoids compounds such as Apigenin, Isoliquiritigenin and Quercetin revealed important anticancer effects [166,167]. Their anticancer mechanisms are essentially related the inducing of apoptosis and/or arrest of cell cycle.

Pancreatic cancer (PC) is one of the most fatal of all cancers [168]. It has been estimated that 458,918 new cases of PC diagnosed in 2018 [169]. Like other types of cancer, several medicinal plants and plant-based products show effects against PC; indeed, several studies (Table 5) have described antitumoral effects of medicinal plants [170]. Berkovich, et al. [171] revealed that the effect of *Moringa oleifera* Lam. leaf extracts on the survival of cultured human pancreatic cancer cells (Panc-1 cells) using flow cytometry analysis. They showed an inhibition of the growth of all pancreatic tested cell lines and inducing an elevation in the sub-G1 cell population of the cell cycle [171]. Moreover, Win et al. [172] reported that the chloroform extract of rhizomes of *Boesenbergia rotunda* (L.) Mansf exhibited important cytotoxic effects against PC cell lines. In addition, studies have identified some molecules with properties against PC [173,174,175]. Indeed, plumbagin, a quinoid isolated from the roots of *Plumbago zeylanica* L., showed important anticancer effect by inducing an apoptosis action and decreasing therefore cell viability of PC cells (Panc-1, BxPC3, and ASPC1). Moreover, in vivo approach has revealed that this compound inhibited the both tumor weight and volume [173]. The use of mimosine, a plant amino acid, subcutaneously growing human PC xenografts in immunosuppressed mice, resulted in significant tumor growth suppression and the sub-G1 fraction, and exerts an apoptotic activity assessed by flow cytometry [174]. Another study, shown that epigallocatechin-3-gallate (EGCG), a polyphenolic compound from green tea, inhibits PC growth and induces apoptosis in human PC cells [175]. A number of studies have shown that the medicinal plant present cytotoxicity towards a number of PC cell lines. The chittagonga extract showed a significant cytotoxic effect on HTB126, Panc-1, Mia-Paca2, and Capan-1 cancer cell lines [176]. Moreover, the dichloromethane-soluble extract of *Angelica pubescens* Maxim. has significant effect against Panc-1 cancer cells [177]. The glycoside (a flavonoid isolated from *Acacia pennata* (L.) Willd.) exhibited a selective cytotoxicity on human pancreatic (Panc-1) [178]. Other volatile compounds such as Betulin and betulinic acid, which are naturally occurring pentacyclic triterpenes, revealed important anticancer effects [179]. Several studies have investigated activity apoptosis in human PC cells induced by medicinal plant and the products of these plants. Inositol hexaphosphate (IP6) decreased cellular growth and increased apoptosis [180]. Sorafenib induced an arrest of PC stem cells (CSC) [181]. On the other hand, triptolide extracted from *Tripterygium wilfordii* Hook. f. significantly increased the apoptotic rates of human PC cells (SW199) [182].

### 3.2. Skin Cancer

Skin cancer is a common disease that accounts for ~4.5% of human cancers, with an average rate of one million new cases each year. This prevalence is increasingly accelerated compared to other cancers. In, the different types of skin cancer cause today a significant morbidity rate, which shows the urgency to identify effective treatments; especially with side effects that are sometimes fatal, conventional treatments used. Today, the search for molecules that may have specific anticancer effects against skin cancer is a promising strategy. Indeed, a number of studies have shown that natural molecules containing in medicinal and aromatic plants have enormous capacity to inhibit tumor growth of skin cancer [183,184,185,186,187,188,189,190]. The cellular mechanisms involved in this anticancer action are numerous, including cell cycle arrest, the induction of apoptosis, and the inhibition of angiogenesis. The following table shows the anticancer properties of natural substances from medicinal and aromatic plants against skin cancer (Table 6). Studies have reported that medicinal plant extracts rich in phenolic compounds showed important anticancer activities against several skin cancer cell lines [187,191]. The polyphenols of *Euphorbia lagascae* Spreng. also showed an important cytotoxicity on melanoma SK-MEL-28 by an arrest of cell cycle at G_2_/M through downregulating cyclins A, E, and B1 expression [187]. Moreover, Tourino et al. [191] reported that the procyanidins of *Pinus pinaster* Aiton induced a cytotoxic effect on the same line (Melanoma SK-MEL-28). Tran et al. [192] demonstrated an antiproliferative activity of *Dracaena angustifolia* (Medik.) Roxb. saponins extracts. On the other hand, numerous isolated compounds from medicinal plants were reported to have antitumor effects on several skin cancer cell lines (Table 6). Certain flavonoids, such as flavone glycoside, exhibited important anticancer effects [183,185,190,193,194,195]. Indeed, Balasubramanian, Narayanan, and Kedalgovindaram and Devarakonda Rama [190] showed that flavone glycoside isolated from *Indigofera aspalathoides* DC. exhibited important cytotoxic effects on several melanoma cell lines (LOX IMVI, MALME-3M, SKMEL-2, SK-MEL-28, SK-MEL-5, UACC-257, and UACC-62). Anastyuk et al. [183] reported that the fucoidans identified in *Fucus evanescens* C. Agardh inhibited colony formation and cell proliferation of SK-MEL-28 and SK-MEL-5 cell lines. Another flavonoid (Galangin) isolated from *Alpinia officinarum* Hance showed cytotoxic effects on B16F10 cell line (murine melanoma cell) via the reducing of the mitochondrial membrane potential. In a remarkable study, Das et al. [195] reported that Apigenin isolated from *Lycopodium clavatum* L. showed an important in vitro cytotoxic effects on human keratinocyte cell line HaCaT. The anticancer activity of Apigenin involved the inhibition of the formation of ROS, and the interference with NF-kB and p38MAPK signaling pathways [195]. The volatile compounds such as terpenes and terpenoids isolated from EOs have also reported as anticancer agents against skin cancer [189,196,197,198]. Linalool (phenolic volatile compound) extracted from the EO of *Satureja thymbra* L. showed cytotoxicity against amelanotic melanoma C32 cell line by inhibiting tumor cells growth [196]. Fouche et al. [197] reported that the Sesquiterpene lactones isolated from *Schkuhria pinnata* (Lam.) Kuntze ex Thell. exhibited remarkable in vitro cytotoxicity on Melanoma UACC-62 cell line. In another study, Darmanin et al. [198] showed that some terpenoids extracted from *Ricinus communis* L. EO, such as 1,8-cineole, camphor and pinene, and caryophyllene, showed important in vitro antiproliferative effects on SK-MEL-28 cells via the inducing of an apoptotic action. Natural alkaloids showed also anticancer properties against skin cancer. This is isoquinoline isolated from *Berberis aristata* DC., which inhibited the growth of human epidermoids carcinoma cell (A-431). 4-Nerolidylcatechol is another natural drug isolated from *Piper umbellatum* L. (synonym of *Pothomorphe umbellate* (L.) Miq.) and has shown important antitumor activity on SK-MEL2, SK-MEL-103, and SK-MEL-147 cell lines. This activity is mediated by the G-1 phase arrest, the inhibition the effect of matrix metalloproteinase MMP-2 and MMP-9 activity, and the loss membrane integrity [184]. Some authors, such as Aggarwal et al. [199] and Niles et al. [200], reported the anticancer activity of Resveratrol (natural compound isolated from *Vitis vinifera* L.) on melanoma (A-375, A-431, and SK-MEL-28). Resveratrol exhibited several anticancer mechanisms, such as the enhanced of the phosphorylation of ERK1/2; the inducing of cell cycle arrest at G1-phase; the inducing of the downregulating of the protein expression of cyclin D1, D2, and E; and also cdk2, cdk4, and cdk6 [199,200].

### 3.3. Brain Cancer

Brain cancer develops in the brain or spinal cord and is categorized into four grades [169], with 296,851 new cases diagnosed in 2018 [201]. The fatality rate due to brain cancer is the highest [202]. Due to their chemical composition, recently, a number of studies have shown that medicinal plant products have anticancer properties against different types of brain cancers. Indeed, numerous compounds from medicinal species, such as *Angelica sinensis* (Oliv.) Diels, *Annona glabra* L., *Bupleurum scorzonerifolium* Wild., and *Bursera microphylla* A. Gray, have shown promising therapeutic effects on brain cancers. Table 7 summarizes the antitumor effects of these medicinal plants on brain cancer [203] have investigated the anticancer property the EO of *Croton regelianus* Müll. Arg. using in vitro and in vivo approaches. The in vitro assay showed that this oil displayed an important cytotoxicity in HL-60 and SF-295 cell lines Moreover, the in vivo study, using sarcoma 180 as a tumor model, demonstrated inhibitions rate of 28.1% and 31.8% for HL-60 and SF-295, respectively [203,204], which demonstrated that both extracts (Leaf oil and berry oil) of *Juniperus phoenicea* L. exhibited important activities against brain cancer human cell lines [204]. However, the ethanolic extract of *Scutellaria baicalensis* Georgi inhibited the cellular growth in recurrent and drug resistant brain tumor cell lines [205]. In another work, the treatment of GBM cells by chloroform extract of *A. sinensis* showed an anticancer effect by displaying the potency in suppressing growth of malignant brain tumor cells by cell cycle arrest and apoptosis. Moreover, the in vitro assay showed that *A. sinensis* triggered both p53-dependent and p53-independent pathways for apoptosis and shrink the volumes of in situ GBM [206].

In addition, studies have identified some molecules with anticancer properties on brain cancer cell lines. Indeed, carvone (volatile compound extracted from several aromatic plants) exhibited remarkable anticancer effects on brain cancer. Its effects are essentially related to increasing in antioxidant level in cancer cells and a potential treatment of brain tumor in primary rat neuron and neuroblastoma (N2a) cells [207]. β-elemene, another volatile compound, extracted from *Curcuma aromatica* Salisb. (synonym of *Curcuma wenyujin*) has shown important anticancer effects on brain cancer. Using an MTT assay and semiquantitative Western blot, the authors demonstrated that this compound inhibited brain carcinomas via the inhibition of U87 cell viability through the activation of the GMFβ signaling pathway. It regulated also the cellular growth, fission, differentiation, and apoptosis [208]. Some flavonoids (fisetin, quercetin, and luteolin) extracted from medicinal plants have reported to be the anticancer agents. Indeed, they inhibited the cancer cell lines growth through the inhibition of protein kinase C (PKC) in brain cancer. This inhibition is mainly effects dose dependent manner and also depending on flavonoid structure [209]. Antioxidant flavonoid compounds, such as delphinidin, pelargonidin, and malvin, are cytotoxic natural agents [210]. Another type of flavonoid (isoflavones), and also an isomer of flavone, was reported as antitumor drugs. Indeed, they decreased the phosphorylation of Akt and eIF4E proteins and rendered U87 cells more sensitive to rapamycin treatment [211].

However, n-butylidenephthalide (BP), which is isolated from the chloroform triggered both p53-dependent and -independent pathways for apoptosis in vitro on glioblastoma multiforme (GBM) cells and suppressed growth of subcutaneous rat and human brain tumors and also, reduced the volume of GBM tumors in vivo [212]. In another study, the authors reported that the effects of BRM270 on glioblastoma stem cells (GSCs) in vitro and GBM recurrence in vivo induced apoptotic cell death and inhibited cell growth [213]. *N*-(4-Hydroxyphenyl) retinamide (4-HPR) has shown activity on two human glioblastoma, T98G and U87MG, cell lines, such as induction of both differentiation and apoptosis in human glioblastoma cells [214]. Moreover, the use of epigallocatechin gallate (EGCG) (bioactive polyphenol in green tea) on GSLCs, which is enriched in human glioblastoma cell line U87, using neurosphere culture has revealed a remarkable inhibited cell viability, neurosphere formation, and also induced apoptosis [215]. The administration of delta-9-tetrahydrocannabinol (Δ9-THC) to GBM cell lines results in a significant decrease in cell viability via a mechanism that appears to elicit G1 arrest due to downregulation of E2F1, cyclin A. Δ9-THC, and cannabidiol acted synergistically to inhibit cell proliferation on the U251 and SF126 glioblastoma cell lines as an essential mediator of cannabinoid antitumoral action [216,217].

### 3.4. Breast Cancer

Cancer is the second leading cause of mortality in developing countries [49], where cancer risk is increasing due to population aging, smoking, and physical inactivity. Several studies have reported the anticancer activity of medicinal plants and their bioactive compounds (Table 8). Liao et al. [218] had reported that the evodiamine—a main constituent of fructus Evodiae—inhibited the proliferation of NCI/ADR-RES cells and caused significant apoptosis with arrest of G2/M cell cycle progression. A study on human breast cancer MDA-231 cells reported that treatment of Sanguinarine isolated from the root of *Sanguinaria canadensis* L. induces remarkable apoptosis [219]. Indeed, this apoptosis may be due to effects on signaling pathways or changes in intracellular proteins [220]. Li et al. [221] studied the action of matrine—a major component derived from *Sophora flavescens* Aiton—on primary and metastatic breast cancer (MCF-7 and 4T1 cells) and they found that it induces cell death in breast cancer cells. Piperine, an alkaloid isolated from *Piper nigrum* L., has been shown to inhibit the growth of 4T1 cells and induce their apoptosis [222].

The exploration of the EOs of different plants revealed that they had anticancer potency against breast cancer [223]. Indeed, *Boswellia sacra* Flueck. EO was effective against T47D, MCF-7, and MDA-MB-231 cells with high cell mortality [224]. Previous studies investigated EOs of several plants, such as *Pulicaria jaubertii* E.Gamal-Eldin [225], *Annona muricata* L. [226], *Cedrelopsis grevei* Baill. & Courchet [227], *Seseli transcaucasicum* Pimenov & Sdobnina (synonym of *Libanotis transcaucasica* Schischk.) [228], *Melissa officinalis* L. [229], and *Sal*via *officinalis* L. [230], have shown a cytotoxic effect on MCF-7 cells. By testing the same cell line, Chen et al. [231] found that they have a high sensitivity towards *Commiphora pyracanthoides* Engl. and *Boswellia carterii* Balf.f. EOs. Also in 2013, [232] have observed an antiproliferative activity of EOs of two species of Thymus against MCF-7 cells [232], and in the same year, *Porcelia macrocarpa* R.E.Fr. EO resulted in viability of SKBr cells [233]. In addition, several authors have investigated the anticancer properties of EOs components, such as carvacrol, which induces apoptosis in MDA-MB-231 cells [234], and citral inducing apoptosis and cell cycle arrest in MCF-7 cells [235]. Similar inhibiting of different phases of cell cycle has also been noted in MCF-7 and MDA-MB-231 cells in response to other phytochemicals [236,237]. This cell arrest is a therapeutic strategy widely used in the prevention of growth and cell division in cancer cells [238]. Moreover, EOs, and their various constituents, present effective anticancer substances by acting on the evolution and expression of the cell cycle [220]. Other studies have focused on evaluating the anticancer activity in vitro of EOs of several medicinal plants against MCF-7 cells by showing their antiproliferative activity [239,240,241].

## 4. Conclusions and Future Remarks

Cancer is one of the leading causes of mortality worldwide and it is imperative to develop novel approaches to treat such diseases. The keystone in cancer combat has been conventional chemotherapy but it is associated with normal cell toxicities. Due to a lack of specificity, conventional cancer treatments often cause severe side effects and toxicities. Generally, natural agents are considered safe while treating or prevention diseases; however, some compounds as flavonoids have shown great potential in the combat against cancer [294]. Plant-derived compounds have a high impact as cancer therapeutic agents both alone or in combination with conventional drugs [295]. Current challenge against cancer is to develop new drugs that include the site specific delivery with low systemic toxicity [296]. A tumor represents a dynamic environment with changes in cell mass, extracellular matrix composition, angiogenic status, among other factors. Promising technologies offer new opportunities to develop new drugs for cancer treatment with lower toxicity associated, but there are still challenges in cancer treatment research: One example is that the formulation of targeted therapies requires the identification of satisfactory molecular targets that have key functions in the growth and survival of cancer cells, and the design and creation of drugs that effectively hit the mark. However, some of the potential targets that have been identified apparently lack places to which an anticancer drug can bind and, therefore, are not susceptible to pharmacological effects. Unfortunately, most of the anticancer FDA-approved drugs and other regulatory agencies have no effect on the overall survival of the cancer patient. Cancer cell lines and animal models are a valuable tool for cancer research but reports indicate that these preclinical models are highly incomplete and not match with results obtained from clinical studies [297]. Finding a way to design drugs that effectively hit the mark is a major challenge. Another example of challenge in cancer treatment is the drug resistance. More research is needed to discover the mechanisms of drug resistance and identify ways to overcome it.

This article focused on natural organic compounds and synthetic derivatives, but there are also natural inorganic compounds with potent anticancer activities. One example is arsenic trioxide (As_2_O_3_) which has been labeled as a poison for years, yet recently have gained importance in the therapy of leukemia and solid cancers [298]. A role change has also been occurred in organic compounds such as artemisinin, a sesquiterpene lactone isolated from the *Artemisia annua* L. (Sweet Wormwood). This compound and its derivatives represent an efficacious antimalarial drug group with an excellent safety profile and, recently, have shown anticancer drug potential [299]. These compounds represent two of the many examples of the power hidden in natural sources and, after many years of research, may become new promising drugs for cancer treatment.

Owing to the explosive rate of new anticancer drug development, there is an urgent need for a synergistic improvement of preclinical studies, clinical trials, pharmacovigilance, and post-marketing surveillance. Several pharmacological agents derived from natural compounds have shown anticancer activity via tumor necrosis factor-related apoptosis-inducing ligand (TRAIL) through direct activation of intrinsic apoptotic pathway or modulation of diverse nonapoptotic pathways to upregulate death receptors [300]. Unfortunately, a lack of robust clinical studies evidence exists to support the in vitro and in vivo results of the widespread use of natural products for chemoprevention and therapy of cancer. Modern technologies and research approaches will uncover the detailed mechanisms of action of the natural products and synthetic derivatives. The development of therapeutic modalities, such as chronotherapy, using natural products and synthetic analogs should be further studied to explore the new cancer treatment avenue.

## Figures and Tables

**Table 1:** Natural antitumor drugs and synthetic analogs.

Antitumor Drug	Chemical Structure	Natural Source/Analog
Paclitaxel	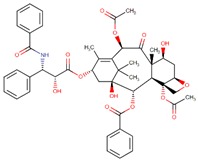	*Taxus brevifolia* Nutt.
Docetaxel	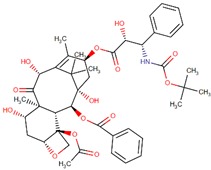	Paclitaxel analog
Cabazitaxel	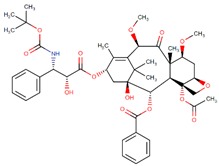	Paclitaxel analog
Camptothecin	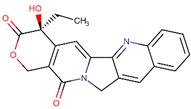	*Camptotheca acuminata* Decne.
Belotecan	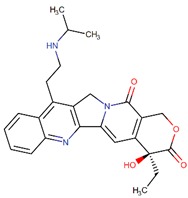	Camptothecin analog
Topotecan	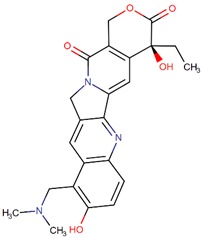	Camptothecin analog
Irinotecan	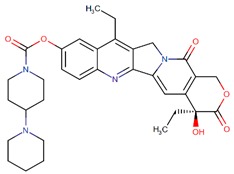	Camptothecin analog
Vinblastine	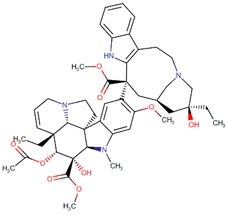	*Vinca rosea* L.
Vincristine	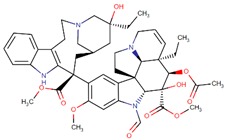	*Vinca rosea* L.
Vindesine	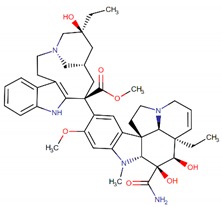	Vincristine analog
Vinorelbine	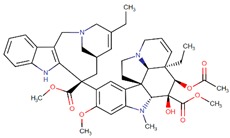	Vincristine analog
Podophyllotoxin	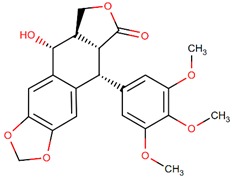	Podophyllum
Etoposide	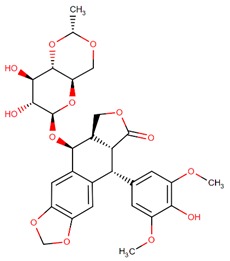	Podophyllotoxin analog
Teniposide	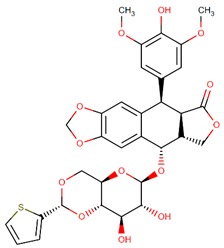	Podophyllotoxin analog
Bleomycin	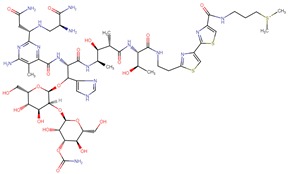	*Streptomyces verticillus*
Dactinomycin	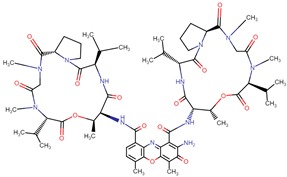	Streptomyces
Doxorubicin	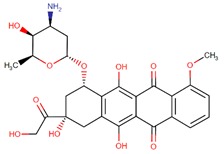	*Streptomyces peucetius* var. caesius
Daunorubicin	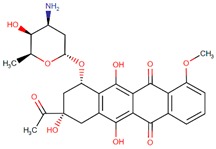	Streptomyces
Epirubicin	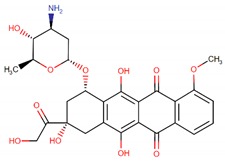	Doxorubicin analog

**Table 2:** Small molecules. Some data were drawn from DrugBank [102].

Ingredient Name Product Name	Chemical Classification	Associated Conditions	Mechanism of Action	Target(s)	FDA Approval
**Cabazitaxel Jevtana**	Organic compound Taxanes and derivatives Diterpenoids	Refractory, metastatic prostate cancer Effective against docetaxel-sensitive and insensitive tumors	Tubulin-based antimitotic	Tubulin alpha-4A chain Tubulin beta-1 chain	June 2010
**Eribulin Halaven**	Organic compound Furopyrans	Metastatic liposarcoma Refractory, metastatic breast cancer Unresectable liposarcoma	Tubulin-based antimitotic	Apoptosis regulator Bcl-2 Tubulin beta-1 chain	November 2010
**VinCRIStine sulfate LIPOSOME Marqibo**	Vinca alkaloids formulated in liposomes (sphingomyelin and cholesterol based)	Relapsed Philadelphia chromosome-negative (Ph-) acute lymphoblastic leukemia Malignant lymphoma Hodgkin’s disease	Tubulin-based antimitotic	Tubulin beta chain Tubulin alpha-4A chain	August 2012
**Rucaparib Rubraca**	Organic compound Indoles and derivatives Indoles	Advanced ovarian cancer	PARPs inhibitor leading to DNA damage, apoptosis, and cell death	Poly(ADP-ribose) polymerase 1 Poly(ADP-ribose) polymerase 2 Poly(ADP-ribose) polymerase 3	December 2016
**Niraparib Zejula**	Organic compound Indoles and derivatives Indoles	Ovarian epithelial cancer Fallopian Tube Cancer Primary Peritoneal Cancer	PARPs inhibitor leading to DNA damage, apoptosis, and cell death	Poly(ADP-ribose) polymerase 1 Poly(ADP-ribose) polymerase 2	March 2017
**Talazoparib Talzenna**	Organic compound Quinolines and derivatives Phenylquinolines	Locally advanced breast cancer Metastatic breast cancer	PARPs inhibitor leading to DNA damage, apoptosis, and cell death	Poly(ADP-ribose) polymerase 1 Poly(ADP-ribose) polymerase 2	October 2018
**Pomalidomide Pomalyst**	Organic compound Isoindoles and derivatives Isoindolines	Refractory multiple myeloma	İnhibition of the proliferation and stimulation of apoptosis. Inhibition of the production of proinflammatory cytokines.	Protein cereblon Tumor necrosis factor Prostaglandin G/H synthase 2	February 2013
**Lenalidomide Revlimid**	Organic compound Soindoles and derivatives Isoindolines	Chronic Lymphocytic Leukemia Mantle Cell Lymphoma Multiple Myeloma	Inhibition of the release of proinflammatory cytokines and increasing the secretion of anti-inflammatory cytokines	Protein cereblon Tumor necrosis factor ligand superfamily member 11 Prostaglandin G/H synthase 2	June 2013
**Erdafitinib Balversa**	Organic compound Organic nitrogen compounds Amines	Locally advanced urothelial carcinoma Metastatic urothelial carcinoma	Inhibition of the enzymatic activity of expressed FGFR1, FGFR2, FGFR3, and FGFR4.	Fibroblast growth factor receptor 1 Fibroblast growth factor receptor 2 Fibroblast growth factor receptor 3 Fibroblast growth factor receptor 4	April 2019
**Lenvatinib Lenvima**	Organic compounds Quinolines and derivatives Quinoline carboxamides	Advanced renal cell carcinoma Locally recurrent radioactive iodine-refractory thyroid cancer Metastatic radioactive iodine-refractory thyroid cancer	İnhibition of the receptor tyrosine kinases leading to suppression of angiogenesis, tumor growth, and cancer progression.	Vascular endothelial growth factor receptor 1/2/3 Fibroblast growth factor receptor 1/2/3/4 Platelet derived growth factor receptor alpha	August 2015

**Table 3:** Activity anticancer of medicinal plants phytochemical compounds on oral cancer.

Plants	Extracts/Molecules	Biological Approach (In Vitro/In Vivo)	Cell Lines Tested	Key Finding/Mechanisms	References
-	Artemisinin and its derivatives	In vitro	YD-10B cell line	Induction of apoptosis	[133]
Amonds	Amygdalin	In vitro	KB cells	cytotoxic and antiproliferative activity	[242]
-	Eugenol	In vitro	HSC-2 cells	Cytotoxic effect	[136]
Cloves oil	Eugenol	In vitro	KB cells	Cytotoxic effect	[137]
-	Eugenol	In vitro	Human oral mucosal fibroblasts	Cytotoxic effect	[138]
*Piper betle* L.	Safrole	In vitro	Human buccal mucosal fibroblasts (BMFs)	Cytotoxic effect	[140]
-	Berberine	In vitro	OC2 and KB cells	- Block cell cycle responses - Cytotoxic effect	[143]
*Thymus vulgaris* L.	Essential oil	In vitro	UMSCC1 cells	Cytotoxic effect	[145]
*Azadirachta indica* A.Juss.	Limonoids	In vivo	Hamster buccal pouch (HBP) carcinogenesis	Inhibition of cell proliferation and induction of apoptosis	[156]
*Alpinia pricei* Hayata	Chalcone	In vitro	HSC-3 cells	- G2/M arrest - induction of apoptosis	[157]
*Pinus densiflora* Siebold & Zucc.	Essential oil	In vitro	YD-8 cells	- Inhibition of proliferation and survival - Induction of apoptosis	[243]
*Artemisia capillaris* Thunb.	Essential oil	In vitro	KB cells	Induction of apoptosis	[150]
*Cinnamomum cassia* (L.) J.Presl	Cinnamaldehyde Essential oil	In vitro	HSC-3 cells	Cell cycle arrest and apoptosis	[244]
*Cryptomeria japonica* (Thunb. ex L.f.) D.Don	Essential oil	In vitro	KB cells	- Induction of apoptosis - Activation of Caspases	[245]
*Solanum spirale* Roxb.	Essential oil	In vitro	KB cells	Cytotoxic effect	[151]
*Sal*via *officinalis* L.	Essential oil	In vitro	UMSCC1 cells	Cytotoxic effect	[146]
*Levisticum officinale* W.D.J.Koch	Essential oil	In vitro	UMSCC1 cells	Cytotoxic effect	[147]
*Elaeagnus angustifolia* L.	Aqueous extract	In vitro	SCC25 cells	- Inhibits of angiogenesis - Induction of differentiation to an epithelial phenotype	[246]
*Neolitsea variabillima* (Hayata) Kaneh. & Sasaki	Essential oil	In vitro	OEC-M1 cells	Cytotoxic effect	[247]
-	Carvacrol	In vitro	OC2 cells	- Induction of apoptosis - Activation of caspase-3	[144]
-	Chios mastic gum extract	In vitro	YD-10B cells	- Inhibition of growth - Induction of apoptosis	[248]
*Saussurea costus* (Falc.) Lipsch.	Methanol extract	In vitro	KB cells	Induction of apoptosis	[249]
*Thymus caramanicus* Jalas	Hydro-ethanolic extractEssential oil	In vitro	KB cells	Cytotoxic effect	[250]
*Artemisia gmelinii* Weber ex Stechm.	Essential oil	In vitro	KB cells	Cytotoxic effect	[149]
*Mentha spicata* L. Associated with *Mentha × rotundifolia* (L.) Huds.	Hexane Extract	In vitro	KB cells	Anti-neoplastic activity	[251]
*Piper betle* L. and *Psidium guajava* L.	Aqueous extract	In vitro	KB cells	Cytotoxic effect	[252]
*Zanthoxylum nitidum* (Roxb.) DC.	Nitidine chloride	In vitro and in vivo	HSC-3 and HSC-4 cells	Decreased cell viability via apoptosis	[253]

**Table 4:** Activity anticancer of medicinal plants phytochemical compounds on gastrointestinal cancer.

Molecules	Biological Approach (in vitro/in vivo)	Cell lines tested	Key Finding/mechanisms	References
*Ganoderma* *lucidum*	In vitro	HCT116	Induced apoptosis in HCT116 and increased caspase-8, caspase-3, and Fas	[159]
Aloe-Emodin	In vitro	SW-620 and HT-29	Suppress cell proliferation in a dose-dependent manner and induced ROS production	[254]
Triterpenes	In vitro	HT-29	Suppresses the proliferation, inhibits tumor growth in the colon carcinoma xenograft model	[160]
Cannabidiol	In vivo	HCT116 mice xenograft	Reduced pre-neoplastic lesions and azoxymethane-induced polyps	[161]
Genistein	In vitro	HT-29	Proapoptotic effect: increases expression of Bax or p21 proteins; inhibits NF-kB and topoisomerase II, in combination with cisplatin inhibits cell growth; and induces apoptosis	[162,163]
Stictic acid	In vitro	HT-29	Moderate anticancer activity and low growth inhibition on nonmalignant cells (MRC-5)	[255]
Apigenin	In vitro	HT-29 and HRT-18	Increases activity of CD26, more in combination with irinotecan	[166]
Quercetin	In vitro	SW-620, HT-29, Caco-2	Sensitizes cells against TRAIL, causing apoptosis, generating of ROS	[167]
Geraniol	In vitro	Caco-2	Increased apoptosis combined with 5-FU	[256]
Lycopene	In vitro	SW480	Acts anti-inflammatory suppresses the expression of PCNA and b-catechins	[164]
*Ginkgo biloba* L. extract	In vitro	HT29	Inhibits progression of the tumor, increases caspase-3 activity, elevates p53 expression, and decreases expression of Bcl-2	[165]

**Table 5:** Activity anticancer of medicinal plants phytochemical compounds on pancreatic cancer.

Plants	Extracts/Molecules	Biological Approach (In Vitro/In Vivo)	Cell Lines Tested	Key Finding/Mechanisms	References
*Plumbago zeylanica* L.	Plumbagin	In vitro	Panc-1, BxPC3, and ASPC1	Induced apoptosis and inhibited the cell viability of PC cells Inhibited the cell invasion of PC cells	[173]
*Plumbago zeylanica* L.	Plumbagin	In vivo		Inhibition of both tumor weight and volume	[173]
*Moringa oleifera* Lam.		In vitro	Panc-1	Inhibited the growth of all pancreatic cell lines Enhanced the cytotoxic effect of cisplatin on Panc-1 cells	[171]
-	Mimosine	In vitro	Xenografts	Inhibited of the cell cycle giving rise to growth arrest in G1-phase	[174]
-	Epigallocatechin-3-gallate	In vitro	Cells in a xenograft model system.	Inhibited the cell growth and induced apoptosis in human pancreatic cancer cells	[175]
-	MK615	In vitro	Panc-1, PK-1, and PK45H	Increased the population of cells in G2/M phase Inhibited the expression of Aurora A and B kinases	[257]
*Acacia pennata* (L.) Willd.		In vitro	Panc-1	Induced a cytotoxic effect	[178]
-	Chittagonga CH_2_Cl_2_	In vitro	Panc-1, Mia-PaCa2, and Capan1	Cytotoxic activity	[176]
*Angelica pubescens* Maxim.	Angelmarin	In vitro	Panc-1	Cytotoxic effect	[177]
	Betulinic	In vitro	EPP85-181P	Cytotoxic effect	[179]
*Boesenbergia rotunda* (L.) Mansf	Chloroform	In vitro	Panc-1	Cytotoxic effect	[172]
-	Cucurbitacin B	In vivo		Inhibited significantly the tumor growth of pancreatic cancer xenografts	[180]
-	Inositol hexaphosphate (IP6)	In vitro	Mia-PaCa et Panc-1	Decreased the cellular growth and increased apoptosis	[22]
-	Apigenin	In vitro	CD18 et S2-013	Decreased glucose uptake and downregulated the GLUT-1 glucose transporter in human pancreatic cancer cells.	[258]
Cruciferous vegetables	Sorafenib	In vivo		Inhibited of angiogenesis Induced of apoptosis	[181]
-	Benzyl isothiocyanate	In vitro	BxPC3, Mia-PaCa2 and Panc-1	Inhibited of cell cycle Activated of apoptotic pathways	[259]
-	L-canavanine	In vitro	Panc-1 and Mia-PaCa2	Synergistic effect with radiation may have clinical potential in the treatment of pancreatic cancer	[260]
-	Apigenin	In vitro	MiaPaca-2, AsPC-1	Induced of apoptosis	[261]
*Tripterygium wilfordii* Hook. f.	Triptolide	In vitro	SW1990	Inhibited the growth of human pancreatic cancer Apoptotic activity	[182]
-	Luteolin	In vitro	SW1990	Induced apoptosis by targeting Bcl-2	[262]
-	Fisetetin	In vitro	Panc-1	Increased autophagy via endoplasmic reticulum stress- and mitochondrial stress-dependent pathways	[263]
-	Resveratrol and quercetin	In vitro	Panc-1	Resveratrol and quercetin affected metastasis in pancreatic cells	[264]

**Table 6:** Activity anticancer of medicinal plants phytochemical compounds on skin cancer.

Plants	Extracts/Molecules	Biological Approach (In Vitro/In Vivo)	Cell Lines Tested	Key Finding/Mechanisms	References
*Piper umbellatum* L.	4-nerolidylcatechol	In vitro	SK-MEL-2, SK-MEL-103, SK-MEL-147	G-1 phase arrest, inhibit the effect of matrix metalloproteinase MMP-2 and MMP-9 activity, loss of membrane integrity	[184]
*Indigofera aspalathoides* DC.	Flavone glycoside	In vitro	Melanoma (LOX IMVI, MALME-3M, SK-MEL-2, SK-MEL-28, SK-MEL-5, UACC-257, UACC-62)	Important cytotoxic effect	[190]
*Fucus evanescens* C.Agardh	Fucoidans	In vitro	SK-MEL-28 and SK-MEL-5	Inhibited colony formation and cell proliferation	[183]
*Alpinia officinarum* Hance	Galangin	In vitro	B16F10 cell line (murine melanoma cell)	Reduced the mitochondrial membrane potential	[185]
*Hamamelis virginiana* L.	Polyphenols	In vitro	SK-MEL-28	Pro-oxidant effects	[265]
*Satureja thymbra* L.	Linalool	In vitro	Amelanotic melanoma C32	Inhibited tumor cell growth (Mechanism Still unknown)	[196]
*Mesua ferrea* L.	Non-polar extract	In vitro	Melanoma SK-MEL-28	Cytotoxicity	[186]
*Euphorbia lagascae* Spreng.	Polyphenol	In vitro	Melanoma SK-MEL-28	Initiated G2/M arrest by downregulating expression of cyclins A, E, and B1.	[187]
*Pinus pinaster* Aiton	Procyanidins	In vitro	SK-MEL-28	Cytotoxicity	[191]
*Vitis vinifera* L.	Resveratrol	In vitro	Melanoma (A-375, A-431, SK-MEL-28)	Enhanced the phosphorylation of ERK1/2. Induced an arresting of the cell cycle at G1-phase. Induced the downregulating of the protein expression of Cyclin D1, D2, and E and also cdk2, cdk4, and cdk6.	[199,200]
*Dracaena angustifolia* (Medik.) Roxb.	Saponins	In vitro	B-16 melanoma cells	Cytotoxicity	[192]
*Schkuhria pinnata* (Lam.) Kuntze ex Thell.	Sesquiterpene lactones	In vitro	Melanoma UACC-62	Cytotoxicity	[197]
*Silybum marianum* (L.) Gaertn.	Silybin	In vitro	Human melanoma SK-MEL-5, SK-MEL-28	Inhibited the expression of Cyclin D1 and caused the cell cycle arrest at G-1 phase. Downregulated the expression of NF-kB, Ap-1 and STAT3. Inhibited the phosphorylation of ERK1/2 and RSK2. Inhibited the activity of MEK1 and MEK2.	[193]
*Silybum marianum* (L.) Gaertn.	Silymarin	In vitro	Human malignant melanoma A375-S2 cells	Increased the expression of cell surface ligand death receptors such as Fas and Fas ligand helped the activation and cleavage of procaspase-8 that cause cell death by apoptosis.	[194]
*Ricinus communis* L.	Terpenoids (monoterpenoids: 1,8-cineole, camphor and pinene, and sesquiterpenoid: caryophyllene)	In vitro	SK-MEL-28 cells	Induced apoptosis	[198]
*Nigella sativa* L.	Thymoquinone	In vitro	Skin cancer	Cytotoxicity	[189]
*Moringa oleifera* Lam.	Extract (silver nanoparticle)	In vitro	A-431 epedermoid carcinoma cell lines	Cytotoxicity	[188]
*Lycopodium clavatum* L.	Apigenin (flavonoid)	In vitro	Human keratinocyte cell line HaCaT	Inhibited the formation of ROS, interfered with NF-kB and p38MAPK signaling pathways	[195]
-	Pyrroloiminoquinone compounds	In vitro	SCC13	Inhibited cancer cell migration and invasion	[266]
-	Citral	In vivo		Inhibited UVB-induced skin carcinogenesis by reducing levels of oxidative stress and proinflammatory cytokines, increasing apoptotic rate in the skin	[267]

**Table 7:** Activity anticancer of medicinal plants phytochemical compounds on brain cancer.

Plants	Extracts/Molecules	Biological Approach (In Vitro/In Vivo)	Cell Lines Tested	Key Finding/Mechanisms	References
*Croton regelianus* Müll.Arg.	Essential oil	In vitro	SF-295	Cytotoxic effects	[203]
*Juniperus phoenicea* L.	Essential oil	In vitro	U251	Cytotoxic effects	[204]
-	Carvone	In vitro	Primary rat neuron and neuroblastoma (N2a) cells	Increased in antioxidant level in primary cells with little potential in treatment of brain tumor	[207]
-	β-Elemene	In vitro	G-422 tumor cells in mice	Inhibited brain carcinomas	[208]
-	Flavonoid	In vitro	Rat brain PKC.	Inhibited the kinase	[209]
*Angelica sinensis* (Oliv.) Diels	Chloroform	In vitro	Glioblastoma multiforme (GBM)	Changed the cell cycle distribution, and induced apoptosis	[206]
*Angelica sinensis* (Oliv.) Diels	Chloroform	In vivo		Reduced the volume of tumor	[206]
-	BRM270	In vitro	GBM	Induced of apoptosis and inhibited cell growth	[213]
-	BRM270	In vivo		Induced of apoptosis and inhibited cell growth	[213]
-	Isoflavones	In vitro	GBM	By its combination with rapamycin, its isoflavones decreased the phosphorylation of Akt and eIF4E proteins, and rendered U87 cells more sensitive to rapamycin treatment	[211]
-	Retinoids	In vitro	T98G	Induced of apoptosis with activation of caspase-8 and cleavage of Bid to truncated Bid (tBid)	[214]
-	Retinoids	In vitro	U87MG	Induced apoptosis with activation of caspase-8 and cleavage of Bid to truncated Bid (tBid)	[214]
-	Epigallocatechin gallate	In vitro	U87	Induced apoptosis via downregulation of p-Akt and Bcl-2	[215]
-	*delta*-9-Tetrahydrocannabinol (Δ^9^-THC)	In vitro	GBM	Decreased cell viability	[216]
-	*delta*-9-tetrahydrocannabinol (Δ^9^-THC)	In vitro	U251 and SF126	Acted synergistically to inhibit cell proliferation	[217]
-	*n*-Butylidenephthalide	In vitro	GBM	Decreased the cell proliferation, and induced apoptotic pathways	[212]
-	*n*-Butylidenephthalide	In vivo		Suppressed the growth of malignant brain tumor cells without inducing cytotoxicity on fibroblast	[212]
*Scutellaria baicalensis Georgi*	Ethanolic extract	In vitro	GBM	Inhibited the cellular growth	[205]
*Phellinus linteus*	Hispolon	In vitro	U87MG	Inhibits cell viability, induced G2/M cell cycle arrest and apoptosis	[268]
-	Aloe-Emodin	In vitro and in vivo	U87MG	Inhibited the cellular growth	[269]

**Table 8:** Activity anticancer of medicinal plants phytochemical compounds on breast cancer.

Plants	Extracts/Molecules	Biological Approach (In Vitro/In Vivo)	Cell Lines Tested	Key Finding/Mechanisms	References
Alpiniae katsumadai	Cardamonin	In vitro	MDA-MB-231 cells	Inhibition of the HIF-1α pathway and modulated cancer cell metabolism	[270]
*Garcinia bracteata* C.Y.Wu ex Y.H.Li	Neobractatin	In vitro	MDA-MB-231 and MCF-7 cells	Prevention of metastasis	[271]
*Tetradium ruticarpum* (A.Juss.) T.G.Hartley	Evodiamine	In vitro and in vivo	NCI/ADR-RES cells	Inhibition of the proliferation of NCI / ADR-RES cells of human breast cancer resistant to adriamycin	[218]
*Sanguinaria canadensis* L.	Sanguinarine	In vivo	- MDA-231 cells - MDA-435S cells	Analyzing changes in expression levels with various antiapoptotic proteins.	[219]
*Sophora flavescens* Aiton	Matrine	In vitro and in vivo	MCF-7 cells and mouse 4T1 breast cancer cell lines	Reduced viability of both types of cells and induction of apoptosis in MCF-7 cells	[221]
*Piper nigrum* L.	Piperine	In vitro and in vivo	4T1 cells	- Induction of apoptosis of 4T1 cells - suppression of primary tumor growth 4T1	[222]
*Curcuma longa* L.	Curcumin	In vivo	MCF-7, MDA-MB-231, BT-474	Inhibition of tumor regression induced by cyclophosphamide.	[22]
-	Carvacrol	In vitro	MDA-MB-231	Induction of apoptosis in cells	[234]
*Boswellia sacra* Flueck.	Essential oil	In vitro	T47D, MCF-7, MDA-MB-231	Suppression of cellular network formation and disruption of spheroid development of breast cancer cells.	[224]
*Nigella sativa* L.	Essential oil	In vitro	MCF-7 cells	Induction of apoptosis in cancer cells.	[272]
*Pulicaria jaubertii* E.Gamal-Eldin	Essential oil	In vitro	MCF-7 cells	Cytotoxic effect	[225]
*Commiphora pyracanthoides* Engl. *Boswellia carterii* Balf.f.	Essential oil	In vitro	MCF-7 cells	Induction of apoptosis in cells	[231]
*Cymbopogon citratus* (DC.) Stapf *Cymbopogon nardus* (L.) Rendle		In vitro	MCF-7 cells	Cytotoxic effect	[273]
*Thymus linearis* Benth. *Thymus serpyllum* L.	Essential oil	In vitro	MCF-7 cells	Antiproliferative activity	[232]
*Porcelia macrocarpa* R.E.Fr.	Essential oil	In vitro	SKBr cells	Cytotoxic effect	[233]
*Annona muricata* L.	Essential oil	In vitro	MCF-7 cells	Cytotoxic effect	[226]
*Cedrelopsis grevei* Baill. & Courchet	Essential oil	In vitro	MCF-7 cells	Cytotoxic effect	[227]
*Seseli transcaucasicum* Pimenov & Sdobnina	Essential oil	In vitro	MCF-7 cells	Cytotoxic effect	[228]
*Melissa officinalis* L.	Essential oil	In vitro	MCF-7 cells	Cytotoxic effect	[229]
*Lycopus lucidus* Turcz. var. hirtus Regel	Essential oil	In vitro	MDA-MB-435S and ZR-75-30 cell lines	Cytotoxic effect	[274]
*Syzygium aromaticum* (L.) Merr. & L.M.Perry	Aqueous extract, ethanolic extract, oil extract	In vitro	MCF-7 and MDA-MB-231	Cytotoxic effect	[275]
*Sal*via *officinalis* L.	Essential oil	In vitro	MCF-7 cells	Cytotoxic effect	[230]
*Mentha spicata* L. *Zingiber officinale* Roscoe *Citrus limon* (L.) Osbeck *Jasminum grandiflorum* L. *Matricaria chamomilla* L. *Thymus vulgaris* L. *Rosa × damascena* Herrm.	Essential oil	In vitro	MCF-7 cells	Cytotoxic effect	[223]
*Laurus nobilis* L. *Origanum syriacum* L. *Origanum vulgare* L. *Sal*via *fruticosa* Mill.	Volatile oil	In vitro	MCF-7 cells	Cytotoxic effect	[276]
*Schinus molle* L. *Schinus terebinthifolia* Raddi	Essential oil	In vitro	MCF-7 cells	Cytotoxic effect	[277]
*Rosmarinus officinalis* L.	Essential oil	In vitro	MCF-7 cells	Antiproliferative activity	[239]
*Schefflera heptaphylla* (L.) Frodin	Essential oil	In vitro	MCF-7 cells	Antiproliferative activity	[240]
*Eucalyptus sideroxylon* A.Cunn. ex Woolls *Eucalyptus torquata* Luehm.	Essential oil, methanolic extract, aqueous extract	In vitro	MCF-7 cells	Antiproliferative activity	[241]
*Schinus molle* L.	Essential oil	In vitro	EMT6 cell lines	Cytotoxic effect	[278]
*Dictamnus albus* L.	Essential oil	In vitro	MCF-7, ZR-75-30 and MDA-MB-435S	Antiproliferative activity	[279]
*Sal*via *officinalis* L. *Sideritis perfoliata* L. *Satureja thymbra* L. *Laurus nobilis* L.	Essential oil	In vitro	MCF-7 cells	Cytotoxic effect	[196]
*Juniperus phoenicea* L.	Essential oil	In vitro	MCF-7 cells	Cytotoxic effect	[204]
*Magnolia ovata* (A.St.-Hil.) Spreng. *Symphyopappus itatiayensis* (Hieron.) R.M.King & H.Rob. *Myrciaria floribunda* (H.West ex Willd.) O.Berg *Psidium cattleianum* Afzel. ex Sabine *Nectandra megapotamica* (Spreng.) Mez	Essential oil	In vitro	MCF-7 cells	Cytotoxic effect	[280]
*Citrus limon* (L.) Osbeck	Essential oil	In vitro	MCF-7 cells	Cytotoxic effect	[281]
*Lavandula stoechas* L.	Essential oil	In vitro	BC1 cells	Cytotoxic effect	[282]
-	Citral	In vitro	MCF-7 cells	Cycle arrest in G2/M phase and apoptosis induction	[235]
-	Eugenol	In vivo	MCF-7 cells	Growth inhibition and apoptosis induction	[283]
-	Galbanic acid	In vitro	MCF-7 and MDA-MB-231 cells	Inhibited proliferation and induced apoptosis	[284]
-	α-Santalol	In vitro	MCF-7 and MDA-MB-231 cells	G2/M phase cell cycle arrest and apoptosis	[236]
-	Piperine	In vivo	MCF-7 cells	Inhibition of self-renewal of breast stem cells	[285]
-	Phytol	in vitro	MCF-7 cells	Cytotoxic activity in a dose-dependent manner	[286]
-	Matrine	In vitro	MDA-MB-231 cell line	Inhibition of proliferation and invasion of cancer cells via the EGF/VEGF-VEGFR1-Akt-NF-κB signaling pathway.	[287]
-	Sanguinarine	In vitro	MDA-MB-231 cells	Inhibition of cell growth and migration of MDA-MB-231 cells	[288]
-	Tetrandrine	In vivo	MCF-7/adr cell lines	inhibition of cell growth in MCF-7 and MCF-7/adr cells	[289]
-	Curcumin	In vitro	MCF-7 cells	Inhibition of proliferative effects of bisphenol A on MCF-7 cells.	[290]
-	Curcumin and citral,	In vitro	MCF-7 and MDA-MB-231 cell lines	Induction of apoptosis and cell cycle arrest at G0/G1 phase in breast cancer cells	[237]
-	Curcumin	In vitro and in vivo	MDA-MB-231 cells	- In vitro: regulation of proliferation and apoptosis in cells - In vivo: inhibition of tumor growth and angiogenesis	[291]
-	Curcumin	In vivo	MDA-MB-435 cells	- Suppress the expression of antiapoptotic, proliferative and metastatic proteins - Strengthen apoptosis.	[292]
-	Curcumin	In vitro and in vivo	MCF-7 and MDA-MB-231	- Induction of apoptosis - inhibition of tumor growth	[293]
